# Activation of the MEK1-CHK2 axis in macrophages by *Staphylococcus aureus* promotes mitophagy, resulting in a reduction in bactericidal efficacy

**DOI:** 10.1186/s10020-025-01274-7

**Published:** 2025-05-29

**Authors:** Xiaohu Wu, Xin Guan, Chubin Cheng, Zhantao Deng, Zeng Li, Yuanchen Ma, Yanjie Xie, Qiujian Zheng

**Affiliations:** 1 Guangdong Cardiovascular Institute, Guangdong Provincial People’s Hospital, Guangdong Academy of Medical Sciences, Southern Medical University, Guangzhou, China; 2 Department of Orthopedics, Guangdong Provincial People’s Hospital (Guangdong Academy of Medical Sciences), Southern Medical University, Guangzhou, 510080 China; 3 Guangdong Provincial Key Laboratory of Bone and Cartilage Regenerative Medicine, Nanfang Hospital, Southern Medical University, Guangzhou, 510515 China; 4 The Second People’s Hospital of Shenzhen City (the First Affiliated Hospital of Shenzhen University), Shenzhen, 518000 China; 5 Department of Health Management Center, The Third Affiliated Hospital, Southern Medical University, Guangzhou, 510515 China

**Keywords:** Osteomyelitis, *S. aureus*, Macrophage mitophagy, MEK1-CHEK2, Bactericidal function

## Abstract

**Background:**

Macrophages, which serve as the frontline defenders against microbial invasion, paradoxically become accomplices in *Staphylococcus aureus* (*S. aureus*)-driven osteomyelitis pathogenesis through poorly defined immunosuppressive mechanisms.

**Methods:**

In this study, we established an *S. aureus* implant-associated femoral infection model treated with MEK1 inhibitors and evaluated the degree of bone destruction and the bacterial load. We subsequently investigated changes in mitochondrial ROS (mtROS) levels, mitophagy activity, phagocytic–killing ability, and CHEK2 mitochondrial translocation in *S. aureus*-activated bone marrow-derived macrophages (BMDMs) following MEK1 inhibitor treatment. Finally, in vivo experiments involving different inhibitor combinations were conducted to assess mitophagy levels and the therapeutic potential for treating osteomyelitis.

**Results:**

Pharmacological inhibition of MEK1 significantly attenuated bone degradation and the pathogen burden in murine models of osteomyelitis, indicating its therapeutic potential. Investigations using BMDMs revealed that blockade of the MEK1-ERK1/2 axis increases mtROS levels by suppressing mitophagy, directly linking metabolic reprogramming to increased bactericidal activity. Mechanistically, inactivation of the MEK1-ERK1/2 pathway restores CHEK2 expression, facilitating its translocation from the nucleus to mitochondria to restore mtROS levels by inhibiting mitophagy. Importantly, in vivo studies confirmed that the MEK1-ERK1/2-CHEK2 axis is pivotal for controlling mitophagy-dependent bone pathology and bacterial persistence during *S. aureus* infection.

**Conclusions:**

We identified a self-amplifying pathogenic loop in which *S. aureus* exploits macrophage MEK1 to hyperactivate ERK1/2, leading to the suppression of CHEK2 expression. This process results in excessive mitophagy and decreased mtROS levels, which impair the bactericidal function and enable uncontrolled osteolytic destruction. These findings redefine MEK1 as a metabolic–immune checkpoint and highlight its druggable vulnerability in osteomyelitis.

**Supplementary Information:**

The online version contains supplementary material available at 10.1186/s10020-025-01274-7.

## Introduction

Osteomyelitis is a debilitating type of infectious inflammation of bone tissue characterized by bone destruction and microbial colonization, predominantly by staphylococcal species (Lew et al. 2004). The management of osteomyelitis continues to pose a significant challenge for orthopedic surgeons. Current therapeutic paradigms, while addressing microbiological clearance, critically overlook the immune metabolic reprogramming of cells in the bone microenvironment that perpetuates microbial persistence (Masters et al. 2019, 2022), resulting in persistent infection and treatment-refractory recurrence (18–25% at the 5-year follow-up) (Trampuz A et al. 2006). Consequently, investigating the mechanisms underlying its occurrence and progression and identifying more effective conservative treatments are of considerable clinical importance.

*Staphylococcus aureus* is the predominant pathogen responsible for osteomyelitis. This bacterium extensively colonizes the pores of bone grafts and forms biofilms, significantly impeding the efficacy of antibiotics (Freiberg et al. 2024). Additionally, *S. aureus* employs various escape mechanisms to evade immune surveillance, even in the presence of effective antimicrobial agents (Zhang et al. 2018). Despite these observations, the precise mechanisms underlying the progression of *S. aureus*-induced osteomyelitis remain elusive. Therefore, further investigations into the molecular pathways governing the onset and development of osteomyelitis are warranted, along with the exploration of novel therapeutic strategies.

Recently, the role of macrophages in bone infections has emerged as a significant research focus (Li et al. 2023). Following invasion by *S. aureus*, macrophages constitute the primary line of defense (Pidwill et al. 2021). However, *S. aureus* not only employs multiple strategies to evade immune surveillance but also has been shown to survive within macrophages (Pidwill et al. 2021; Flannagan RS et al. 2018). Moreover, during the later stages of infection, macrophages are at risk of depletion, leading to uncontrolled infection progression (Hur DG et al. 2021). Consequently, elucidating the molecular mechanisms underlying macrophage function in *S. aureus* infection is crucial.

Our previous studies demonstrated that MEK1/2 signaling in macrophages activated by *S. aureus* infection is a critical mediator of the development of osteomyelitis in mice. The combination of specific inhibitors with sensitive antibiotics has shown promise in treating murine osteomyelitis (Jin et al. 2024). However, MEK1/2 signaling is ubiquitous and plays a vital role in normal cell growth and development (Wu PK et al. 2020). Blockade of this pathway may result in a range of adverse effects. Notably, MEK1 and MEK2 are two isoforms involved in MEK1/2 signaling with approximately 80% functional overlap (Catalanotti et al. 2009). However, their distinct roles in infection remain to be elucidated. Therefore, with the aim of developing a safer and more effective treatment strategy for osteomyelitis, we hypothesize that MEK1, rather than MEK2, plays a pivotal role in the progression of osteomyelitis following *S. aureus* infection of macrophages.

In this study, our findings revealed that *S. aureus* infection significantly activated MEK1 but not MEK2 in macrophages. The inhibition of MEK1 markedly enhanced the bactericidal activity of macrophages and increased mtROS production. Specifically, we observed that MEK1 activation in macrophages enhanced mitophagy by downregulating CHEK2 expression, thereby suppressing mtROS release. Consequently, this research aims to elucidate the critical role of overactivated MEK1 signaling in macrophages during *S. aureus*-induced osteomyelitis, which may represent a potential novel therapeutic target for osteomyelitis.

## Materials and methods

### Bacterial culture

The *Staphylococcus aureus* strain used in this study was isolated from a clinical case of chronic osteomyelitis in a patient whose pathogen exhibited sensitivity to gentamicin in antimicrobial susceptibility testing (Lin Y et al. 2021). Monoclonal colonies of *S. aureus* were selected from infected tissue samples and expanded through large-scale clonal replication. The bacteria were aliquoted and cryopreserved at −80 °C. For each experiment, a single aliquot was thawed and used without refreezing. Bacterial suspensions were cultured on tryptic soy agar (TSA) plates, and individual *S. aureus* colonies were inoculated into tryptic soy broth (TSB) and incubated for 16–18 h at 200 rpm under aerobic conditions. The bacterial pellets were collected by centrifugation, resuspended in PBS, and adjusted to a concentration of 10^8^ CFUs/mL based on an optical density (OD600) measurement of 0.5. The suspension was further diluted with PBS to a working concentration of 10^6^ CFUs/mL.

### Animal model and treatments

All C57BL/6 mice used in this study were provided by the Experimental Animal Center of Southern Medical University with valid “Experimental Animal Quality Certificates”. The animals were housed in the Experimental Animal Center of Southern Hospital, Southern Medical University. Specific pathogen-free (SPF) mice were maintained under controlled conditions (temperature: 20–25 °C; relative humidity: 50 ± 5%) with a 12-hour light/dark cycle. All the mice had free access to food and water in accordance with the “Experimental Animal Management Regulations of Southern Hospital, Southern Medical University”.

Our laboratory has successfully established a stable and effective implant-associated *S. aureus* osteomyelitis model through repeated validation (Lin Y et al. 2021). In this study, 10–12-week-old male mice were anesthetized via an intraperitoneal injection of a tribromoethanol working solution (250 mg/kg). Following adequate anesthesia, the right femoral greater trochanter was surgically exposed through a longitudinal skin incision. A unicortical drill hole was created using a 1 ml syringe needle, followed by medullary cavity reaming and the implantation of a 2 mm × 1.0 mm medical device into the intramedullary canal. Subsequently, 2 µL of the bacterial suspension (1 × 10⁶ CFUs/mL) was injected using a microsyringe, followed by sealing with bone wax and layered suturing. The control group received an equivalent volume of sterile PBS. Postoperative care included normal feeding. Starting from postoperative Day 1, both the control and infection groups received gentamicin treatment (20 mg/kg, intraperitoneal injection). The animals were sacrificed at 3, 7, and 14 days after surgery for tissue collection.

Inhibitor treatment groups: The establishment of the 14-day implant-associated *S. aureus* osteomyelitis model followed identical procedures. Animals were randomly divided into groups and received the following treatments beginning on postoperative Day 7: GDC-0973 (2 mg/kg, i.p., #HY-13064, MCE, USA), GDC-0973 + BML-277 (2 mg/kg, i.p., #HY-13946, MCE, USA), and LY3214996 (1 mg/kg, i.p., #HY-101494, MCE, USA) + BML-277. The control group received an equivalent volume of the same solvent. All the animals were sacrificed at 14 days after surgery for tissue collection.

### Micro-CT

Postoperative murine femoral specimens were fixed with 4% paraformaldehyde for 24 h and subsequently preserved in 70% ethanol. Prior to scanning, the samples were immersed in phosphate-buffered saline (PBS) for 6 h to remove the residual fixatives. High-resolution micro-CT scanning was performed via a SkyScan system (Bruker) with the following parameters: 50 kV voltage, 200 µA current, and 10 μm/voxel resolution. The scan region was centered on the implant, covering 1.5 mm proximal and distal to the device (total scan length: 3 mm, ~ 300 slices), to evaluate cortical bone loss and reactive new bone formation following infection. Using identical parameters, the distal femoral trabecular bone region was additionally scanned to assess bone mineral density (BMD) and trabecular microstructure indices. Quantitative analyses were conducted using the SkyScan 1176 software suite (CTAn, CTvox, DataViewer) to measure the bone volume/tissue volume (BV/TV), trabecular thickness (Tb.Th), trabecular number (Tb.N), trabecular separation (Tb.Sp), and trabecular pattern factor (Tb.Pf).

### Bacterial burdens in infected femurs

The mice were anesthetized with tribromoethanol (250 mg/kg) and euthanized via cervical dislocation at 14 days after infection. The implanted femurs were dissected free of soft tissues, and the intramedullary implants were carefully removed and homogenized in 1 mL of PBS using a sterile tissue grinder. Serial tenfold dilutions of the homogenate were plated on TSA plates to quantify the *S. aureus* burden. Following a 12-hour incubation at 37 °C, bacterial colonies were imaged and enumerated using ImageJ software (Version 1.52a, NIH), with viable counts reported as colony-forming units per milliliter (CFUs/mL) of the original homogenate.

### Histological analyses

Following euthanasia, femoral specimens were collected and subjected to hardware removal. The samples were fixed with 4% paraformaldehyde for 48 h and then decalcified in a 10% ethylenediaminetetraacetic acid (EDTA) solution for 7–10 days prior to paraffin embedding. Serial 10-µm-thick paraffin sections were prepared and stained with hematoxylin and eosin (H&E) using standard protocols. Histopathological scoring was performed according to the established criteria described previously, which evaluated four osteomyelitis-related pathological features: acute intraosseous inflammation, chronic intraosseous inflammation, periosteal inflammation and bone necrosis. Each parameter was graded on a 0–4 point scale.

### Immunohistochemistry

Paraffin sections (5 μm thick) were deparaffinized in xylene and rehydrated through a graded ethanol series. Antigen retrieval was performed by heating in 10% Tris-EDTA buffer (pH 8.0) at 95 °C for 15 min. Endogenous peroxidase activity was blocked with 3% H_2_O_2_ for 20 min, followed by blocking with 5% BSA. The sections were incubated overnight at 4 °C with a rabbit anti-p-MEK1 monoclonal antibody (1:100, #ET1612-40, HUABIO, China), while negative controls received PBS instead of the primary antibody. After 1 h of incubation at room temperature with an HRP-conjugated goat anti-rabbit secondary antibody (1:500, MCE, USA), the DAB chromogen was applied for 30–90 s while monitoring color development under a microscope, followed by hematoxylin counterstaining. Images of whole sections were acquired using a NanoZoomer scanner. The quantitative analysis was performed by randomly selecting five 200 × 200 μm fields and applying fixed DAB signal thresholding (brown channel) to calculate the p-MEK1-positive signal intensity (1/mm²).

### Flow cytometry

Following euthanasia, the surgical-side femurs were aseptically isolated and flushed with 1 mL of ice-cold PBS containing 2% fetal bovine serum (FBS) to obtain single-cell suspensions from the bone marrow. The suspensions were filtered through a 70 μm cell strainer (#15–1070, Shandong BioLix Co., China) and treated with ACK lysis buffer (#CS0001, Beijing LaiGene Co., China) for 5 min at room temperature to lyse the erythrocytes. Cell viability (> 95%) was confirmed by trypan blue exclusion, and the cell density was adjusted to 1 × 10^6^ cells/mL.

Antibody Staining Protocol: For Fc receptor blockade, the cells were preincubated with an anti-mouse CD16/CD32 monoclonal antibody (#01319, BioLegend, USA, 1:50) in 100 µL of staining buffer for 10 min. For surface marker staining, the following antibody cocktail was added and incubated on ice for 30 min: CD11b-BV421 (#101235, BioLegend, 1:80), Ly6G-PerCP (#127653, BioLegend, 1:80), F4/80-BV711 (#123147, BioLegend, 1:40), Gr-1-FITC (#108405, BioLegend, 1:100), and the Mitophagy Detection Kit (#MD01, DOJINDO). Intracellular staining: After fixation/permeabilization (Cytofix/Cytoperm Kit, BD), the cells were incubated with a primary antibody against phospho-MEK1 (#ET1612-40, HUABIO, 1:50) for 20 min on ice, followed by an incubation with a secondary antibody (#34206ES60, Yeasen, China, 1:100) for 20 min. The samples were analyzed on a BD LSRII flow cytometer (3-laser configuration, BD Biosciences, USA). The flow cytometry analysis using FlowJo v10.8 involved sequential gating: live singlets (FSC-A/SSC-A → FSC-H/FSC-W) were selected, followed by the identification of macrophage (CD11b + F4/80+) and neutrophil (CD11b + Ly6G+) populations within the viable cell population.

### Cell culture

All in vitro studies were conducted using primary bone marrow-derived macrophages (BMDMs) to obtain high-level experimental evidence. Femurs and tibiae from 6–8-week-old C57BL/6 mice were aseptically isolated after euthanasia, followed by bone marrow flushing with L929-conditioned medium (containing macrophage colony-stimulating factor [M-CSF]) using a 1 mL syringe. The cell suspension was filtered through a 70 μm strainer, centrifuged (300 ×g, 5 min), and treated with ACK lysis buffer (5 min, RT) to remove erythrocytes. After washing, the cells were resuspended in complete RPMI-1640 medium (10% FBS + 1% penicillin/streptomycin + 30% L929 supernatant) and seeded at 2 × 10^6^ cells/mL in 6-well plates.

### SiRNA transfection of BMDMs

Each tube of lyophilized siRNA powder was resuspended in 125 µL of double-distilled water (ddH₂O) to prepare a 20 µM stock solution. A total of 2 × 10⁵ BMDM cells were seeded per well in a 24-well plate and incubated for 24 h before transfection. For transfection, 1.25 µL of the siRNA stock solution (si-MEK1 or si-MEK2, #sc-35904 or #sc-35906, Santa Cruz Biotechnology, USA) was diluted in 50 µL of Opti-MEM and mixed thoroughly by pipetting (Solution A). Separately, 1 µL of Lipofectamine 3000 was diluted in 50 µL of Opti-MEM and mixed gently (Solution B). Solution B was slowly added to Solution A, the mixture was pipetted gently, and the mixture was incubated at room temperature for 30 min. The complex was then added dropwise to the cells, and the plate was gently swirled to ensure an even distribution. After 6 h of coincubation, the medium was replaced with 1 mL of fresh complete medium containing antibiotics. Culturing continued for 48 h before proceeding with subsequent experiments performed promptly. The siRNA sequences used were as follows: si-MEK1, sense 5′-GGA ACA UGU CUG CUA CUA U dTdT-3′, antisense 5′-AUA GUA GCA CAA UGU UCC dTdT-3′; and si-MEK2, sense 5′-GCA UCA AGU UCG AGU UCA A dTdT-3′, antisense 5′-UUG AAC UCG AAC UUG AUG C dTdT-3′.

### Western blotting

Femurs were isolated from a 7-day murine model of implant-associated *S. aureus* osteomyelitis for the bone tissue analysis. For in vitro studies, BMDMs were pretreated with 10 µM GDC-0973 (#HY-13064, MCE, USA), 5 µM LY3214996 (#HY-101494, MCE, USA) or siRNAs and subsequently stimulated with *S. aureus* at a multiplicity of infection (MOI) of 10 for 1 h prior to sample collection. Tissue or cell lysates were prepared using RIPA lysis buffer (#89901, Thermo Scientific, USA) supplemented with protease (#HY-K0010, MedChemExpress, USA) and phosphatase inhibitors (#HY-K0023, MedChemExpress, USA). The lysate was centrifuged to collect the supernatant, and the protein concentration was quantified using a Pierce BCA Protein Assay Kit (#23225, Thermo Fisher Scientific, USA). After denaturation with SDS‒PAGE loading buffer (#GC-CW0227 A, SANTAIBIO, USA) by boiling, the proteins were separated via SDS‒PAGE (#PG212, Yeasen Biotechnology, China) and transferred to PVDF membranes (#IB24001, Invitrogen, USA) using a vertical electrophoresis transfer system (#1658033, Bio-Rad, USA). The membranes were blocked with 5% skim milk in TBST for 30 min at room temperature, followed by an overnight incubation at 4 °C with the primary antibodies (p-MEK1 #ET1612-40, p-MEK2 28955-1-AP, p-ERK1/2 #ET1610-13, MEK1 #ET1603-20, MEK2 #ET1612-6, ERK1/2 #ET1601-29, LC3B #ET1701-65, p62 #HA721171, and GAPDH #R1210-1; HUABIO China or Proteintech USA; diluted 1:1,000–1:5,000) and a 1-hour incubation at room temperature with an HRP-conjugated secondary antibody (#HA1006, HUABIO, 1:10,000, China). After washes with TBST, the protein bands were visualized via a chemiluminescence imaging system (Guangzhou Aivie Biotechnology, China) with Western Lightning Plus ECL reagent (#NEL105001EA, PerkinElmer, USA). Band intensities were analyzed via ImageJ software (version 1.52a, NIH) and normalized to that of GAPDH, which was used as the loading control.

### Bacterial phagocytosis and killing capability

BMDMs were seeded in 24-well plates at a density of 2 × 10⁵ cells/well and cultured for 7 days. The medium was replaced with antibiotic-free medium, and the cells were pretreated for 24 h with 10 µM GDC-0973 (#HY-13064, MCE, USA), 5 µM LY3214996 (#HY-101494, MCE, USA), 5 µM Mdivi-1 (#HY-15886, MCE, USA), or si-MEK1. Subsequently, cells were incubated with *S. aureus* at an MOI of 10 for 1 h to assess phagocytosis, followed by a 1-hour treatment with lysostaphin (20 µg/mL) and gentamicin (50 µg/mL) to eliminate extracellular bacteria. The cells were lysed with 0.1% Triton X-100, serially diluted, and plated on TSB agar overnight at 37 °C; the phagocytic efficiency was quantified by colony-forming unit (CFU) counts. For the intracellular bacterial killing assays, after extracellular bacterial clearance, the cells were further cultured for 12 h with or without inhibitors/siRNAs, lysed, and CFUs were enumerated. The killing rate was calculated as [(phagocytosed CFUs − surviving CFUs)/phagocytosed CFUs] × 100% to evaluate the role of MEK1/2 signaling in macrophage antimicrobial function.

### Transmission electron microscopy

BMDMs were plated in 24-well culture plates at 1 × 10⁵ cells/well and differentiated for 7 days. The cells in selected wells were pretreated for 24 h with either 10 µM GDC-0973 (#HY-13064, MCE, USA) or 5 µM LY3214996 (#HY-101494, MCE, USA), whereas the control wells received no inhibitor. The culture medium was then aspirated directly from the cells, and an ample amount of prechilled electron microscopy-grade fixative was quickly added for fixation for 1 h (to ensure complete submersion of the cells). A cell scraper held at a 45° angle was subsequently used to swiftly scrape the cells in one direction, which were then transferred to a 1.5 ml EP tube. The mixture was centrifuged at 1000 rpm for 5 min, the supernatant was discarded, 1 ml of electron microscopy fixative was added, the mixture was centrifuged again, and the supernatant was removed (the ideal cell pellet size should be approximately half the size of a mung bean). One milliliter of fresh electron microscopy fixative was added, and the mixture was then stored at 4 °C for continued fixation and preservation. The ultrastructural analysis was performed using a field-emission transmission electron microscope (FEI Tecnai G2 F20 S-TWIN). Particle colocalization was quantified using ImageJ software (v1.52a, NIH).

### Evaluation of MtROS levels and mitophagy

BMDMs were seeded into confocal dishes at a density of 5 × 10⁵ cells per well and cultured for 7 days to allow maturation before mtROS levels and mitophagy were assessed. After maturation, the BMDMs were pretreated with 10 µM GDC-0973 (#HY-13064, MCE, USA), 10 µM BML-277 (#HY-13946, MCE, USA), 5 µM LY3214996 (#HY-101494, MCE, USA), or an equivalent volume of solvent, followed by stimulation with *S. aureus* (MOI = 10) for 1 h. Extracellular bacteria were eliminated by replacing the medium with lysostaphin (20 µg/mL) and gentamicin (50 µg/mL). Subsequently, fresh medium containing 1% penicillin/streptomycin was added, and the cells were incubated until the designated time points were reached. For mtROS detection, the cells were costained with MitoSOX Red (#M36008, Invitrogen, USA) and MitoTracker Green FM (#M7514, Invitrogen, USA). Mitophagy was assessed using LysoTracker Red (#GC19882-50, GLPBIO, USA) in combination with MitoTracker Green FM. After bacterial clearance, the cells were washed with serum-free RPMI-1640 and incubated with staining solutions containing either 5 µM MitoSOX Red and 200 nM MitoTracker Green FM (for mtROS) or 5 µM LysoTracker Red and 200 nM MitoTracker Green FM (for mitophagy) at 37 °C in the dark for 15–25 min. Fluorescence imaging was performed using a confocal laser microscope (LSM980, Carl Zeiss, Germany, Objective Plan-Apochromat 20X/0.8 M27) with appropriate excitation/emission filters. Images of five random fields per experimental group were captured, and the experiments were independently repeated five times. MtROS levels were quantified as the ratio of foci in which MitoSOX Red (red foci) to MitoTracker Green (green foci) colocalized. Mitophagy was evaluated by analyzing the colocalization of LysoTracker Red (red foci) and MitoTracker Green (green foci). Imaging data were processed for particle colocalization analysis using ImageJ software (version 1.52a, NIH).

### Detection of CHEK2 mitochondrial translocation

BMDMs were pretreated with 10 µM GDC-0973 (#HY-13064, MCE, USA) or 5 µM LY3214996 (#HY-101494, MCE, USA) as described above. After mitochondrial staining with MitoTracker Green FM (#M7514, Invitrogen, USA), the cells were fixed with 4% paraformaldehyde for 10 min. Following PBS washes, the cells were incubated in blocking buffer at room temperature in the dark for 1 h and then incubated with a rabbit anti-checkpoint kinase 2 (CHEK2) primary antibody (#13954-1-AP, Proteintech, USA) for 2 h at room temperature in the dark. After washes with 0.1% PBST, the cells were incubated with CoraLite594-conjugated goat anti-rabbit IgG (H + L) (#SA00013-4, Proteintech, USA) for 2 h. DAPI was used to label the nuclear DNA. Images were acquired using a fluorescence microscope (Zeiss LSM980, Germany). The mitochondrial translocation of CHEK2 was assessed by analyzing the colocalization of CHEK2 (red foci) and MitoTracker Green (green foci). The imaging data were processed using ImageJ software (version 1.52a; National Institutes of Health, USA) for the particle colocalization analysis.

### Statistical analysis

Statistical analyses were performed via GraphPad Prism 8.0 (GraphPad, USA). For comparisons between two groups, parametric data were analyzed by Student’s *t* test, whereas nonparametric data were assessed using the Mann‒Whitney *U* test. For multigroup comparisons, one-way analysis of variance (ANOVA) with Tukey’s *post hoc* test were applied. The data are presented as the means ± SEMs. A p value < 0.05 was considered statistically significant.

## Results

### The phosphorylation of MEK1 in macrophages is increased in the femurs of mice with *S. aureus*-induced osteomyelitis

**Fig. 1:**
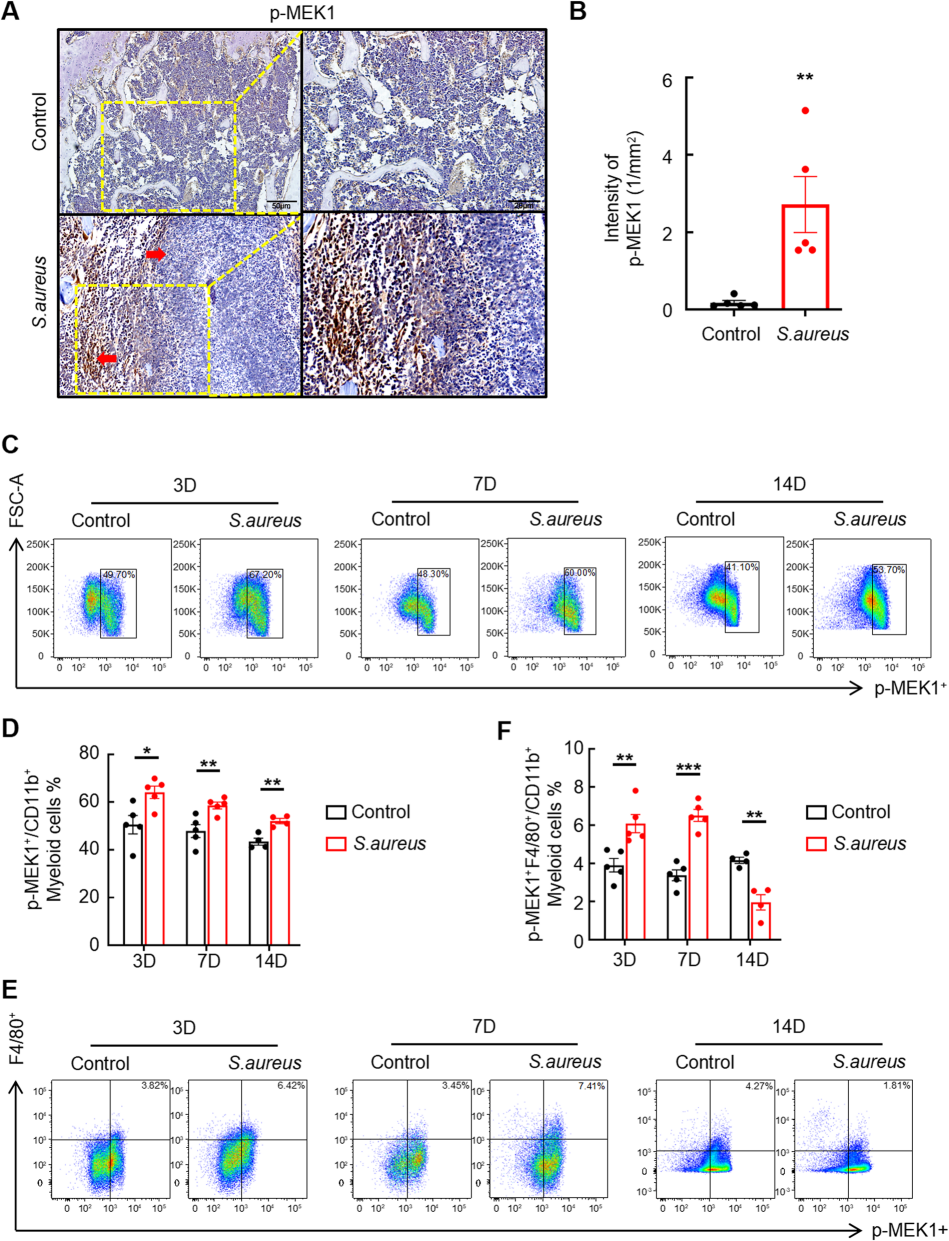
The phosphorylation of MEK1 in macrophages is increased in the femurs of mice with *S. aureus*-induced osteomyelitis. **A** and **B** Representative images (**A**) and quantification (**B**) of immunohistochemical staining for p-MEK1 in the implanted femurs of mice with *S. aureus*-induced osteomyelitis compared with those of control mice. The left-pointing red arrows mark regions with p-MEK1-positive staining, whereas the right-pointing red arrows highlight abscess areas. Scale bars, 50 μm–20 μm. **C**–**F** Representative images (**C** and **E**) and quantification (**D** and **F**) of flow cytometry data for the proportions of CD11b^+^p-MEK1^+^ and CD11b^+^p-MEK1^+^F4/80^+^ cells in the bone marrow of mice with *S. aureus*-induced osteomyelitis and control mice at 3, 7, and 14 days after surgery. *n* = 5 mice/group, *p < 0.05, **p < 0.01, and ***p < 0.001

Our previous study identified a crucial role for MEK1/2 in *S. aureus*-induced osteomyelitis that is characterized by the overactivation of MEK1/2 signaling in macrophages (Jin et al. 2024). We further elucidated the mechanisms underlying osteomyelitis progression mediated by MEK1/2 signaling by measuring the levels of phosphorylated MEK1 in the femurs of *S. aureus*-infected mice after 14 days via immunohistochemistry (Lin Y et al. 2021). Compared with the control group, the level of p-MEK1 in the bone marrow cavity of *S. aureus*-infected mouse femurs was markedly increased (Fig. 1A), and the statistical analysis revealed a significant increase in the p-MEK1 intensity in the infected group (Fig. 1B). Notably, high-intensity p-MEK1 signals were predominantly localized around abscesses, regions rich in immune cells such as macrophages and neutrophils.

We analyzed the proportions of the p-MEK1^+^/F4/80^+,^ p-MEK1^+^/Ly6G^+^, and p-MEK1^+^/Gr-1^+^ cell populations in the medullary cavity of infected mice at specific time points using flow cytometry to determine which immune cells contained phosphorylated MEK1. Consistent with our previous findings, the proportion of p-MEK1^+^ cells among CD11b^+^ myeloid cells was significantly higher at 3, 7, and 14 days postinfection than in the control groups (Fig. 1C and D). Specifically, the proportion of p-MEK1^+^/F4/80^+^ cells among CD11b + myeloid cells increased markedly at 3 and 7 days postinfection, whereas the opposite trend was observed at 14 days between the *S. aureus*-infected and control groups (Fig. 1E and F). This discrepancy is likely attributable to the severe infection on Day 14, which resulted in macrophage depletion. In addition, no significant differences were observed in the proportions of p-MEK1^+^/Ly6G^+^ or p-MEK1^+^/Gr-1^+^ cells among the CD11b^+^ myeloid cells at any time point between the infected and control groups (Figure S1 A and S1B). Collectively, these results suggest that the sustained activation of phosphorylated MEK1 signaling in macrophages likely plays a critical role in *S. aureus*-induced femoral osteomyelitis in mice.

### Macrophage-specific MEK1, but not MEK2, drives *S. aureus*-induced activation of the MEK1/2-ERK1/2 signaling axis

**Fig. 2:**
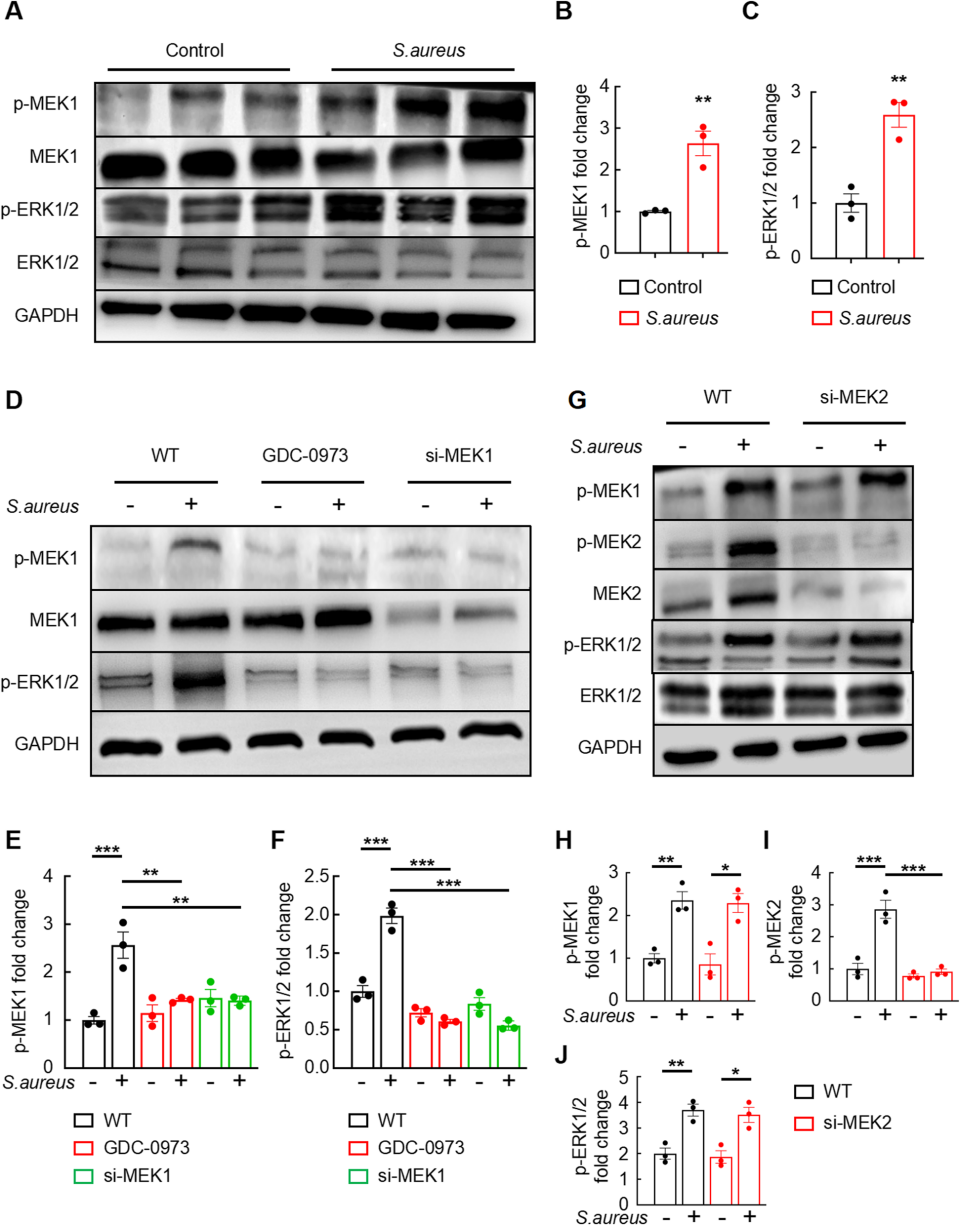
MEK1, but not MEK2, in macrophages drives the *S. aureus*-induced activation of the MEK1/2-ERK1/2 signaling axis. **A**–**C** Representative images (**A**) and quantification (**B** and **C**) of western blots showing p-MEK1 and p-ERK1/2 protein levels in the implanted femora of mice with *S. aureus*-induced osteomyelitis compared with those in the control mice. *n* = 3 mice/group. **D**–**F** Representative images (**D**) and quantification (**E** and **F**) of western blots showing p-MEK1 and p-ERK1/2 protein levels in *S. aureus*-infected BMDMs treated with GDC-0973 or the MEK1-targeting siRNA. **G**–**J** Representative images (**G**) and quantification (**H**–**J**) of western blots showing p-MEK1, p-MEK2 and p-ERK1/2 protein levels in *S. aureus*-infected BMDMs treated with the MEK2-targeting siRNA. *n* = 3 samples/group. *p < 0.05, **p < 0.01, and ***p < 0.001

The MEK1/2 complex comprises two subunits, MEK1 and MEK2. Our findings suggest that MEK1 signaling in macrophages plays a critical role in *S. aureus*-induced osteomyelitis. We validated this hypothesis by conducting a series of experiments via western blot analysis. First, we compared the protein levels of p-MEK1, MEK1, p-ERK1/2, ERK1/2, and GAPDH in bone marrow extracts from the control and *S. aureus*-infected groups. The results revealed a significant increase in p-MEK1 levels in the *S. aureus*-infected group (Fig. 2A and B), which was consistent with our previous findings. Additionally, we observed the significant upregulation of p-ERK1/2 signaling in the bone cavities of *S. aureus*-infected mice compared with control mice (Fig. 2A and C), confirming that *S. aureus* infection activated the MEK1-ERK1/2 signaling pathway.

We subsequently examined whether *S. aureus* could stimulate the MEK1 pathway in primary BMDMs. A predominant increase in p-MEK1 and p-ERK1/2 levels was observed in BMDMs from the wild-type control in response to *S. aureus* infection (Fig. 2D). A quantitative analysis of the western blot bands for p-MEK1 and p-ERK1/2 revealed substantial increases in their levels in the wild-type experimental group (Fig. 2E and F). Furthermore, the western blot analysis indicated that *S. aureus* infection, treatment with the MEK1 inhibitor GDC-0973, and MEK1 knockdown led to marked reductions in p-MEK1 and p-ERK1/2 levels (Fig. 2D). An additional analysis confirmed that GDC-0973 and MEK1 knockdown significantly decreased the levels of the p-MEK1 and p-ERK1/2 proteins compared with those in the wild-type group stimulated with *S. aureus* (Fig. 2E and F).

We investigated p-MEK1, p-MEK2, MEK2, p-ERK1/2, ERK1/2, and GAPDH protein levels in MEK2 knockdown-treated BMDMs stimulated with *S. aureus* to exclude the role of MEK2 signaling in *S. aureus* infection of macrophages. Consistent with our expectations, the levels of MEK1, MEK2, and ERK1/2 phosphorylation were dramatically increased in the wild-type groups upon *S. aureus* infection (Fig. 2G and J). Even in MEK2-knockdown BMDMs, p-MEK1 was rapidly activated by *S. aureus* stimulation, whereas the level of p-MEK2 showed the opposite trend (Fig. 2G and I). Moreover, the levels of the p-ERK1/2 proteins in MEK2-knockdown BMDMs stimulated with *S. aureus* were dramatically higher than those in the BMDMs treated with the isotype control (Fig. 2G and J). Overall, we determined that MEK1, rather than MEK2, was required for the phosphorylation of proteins in the ERK1/2 signaling pathway in macrophages during *S. aureus* infection.

### The inhibition of MEK1 signaling attenuates *S. aureus*-induced osteomyelitis by reducing bone destruction and the bacterial burden

**Fig. 3:**
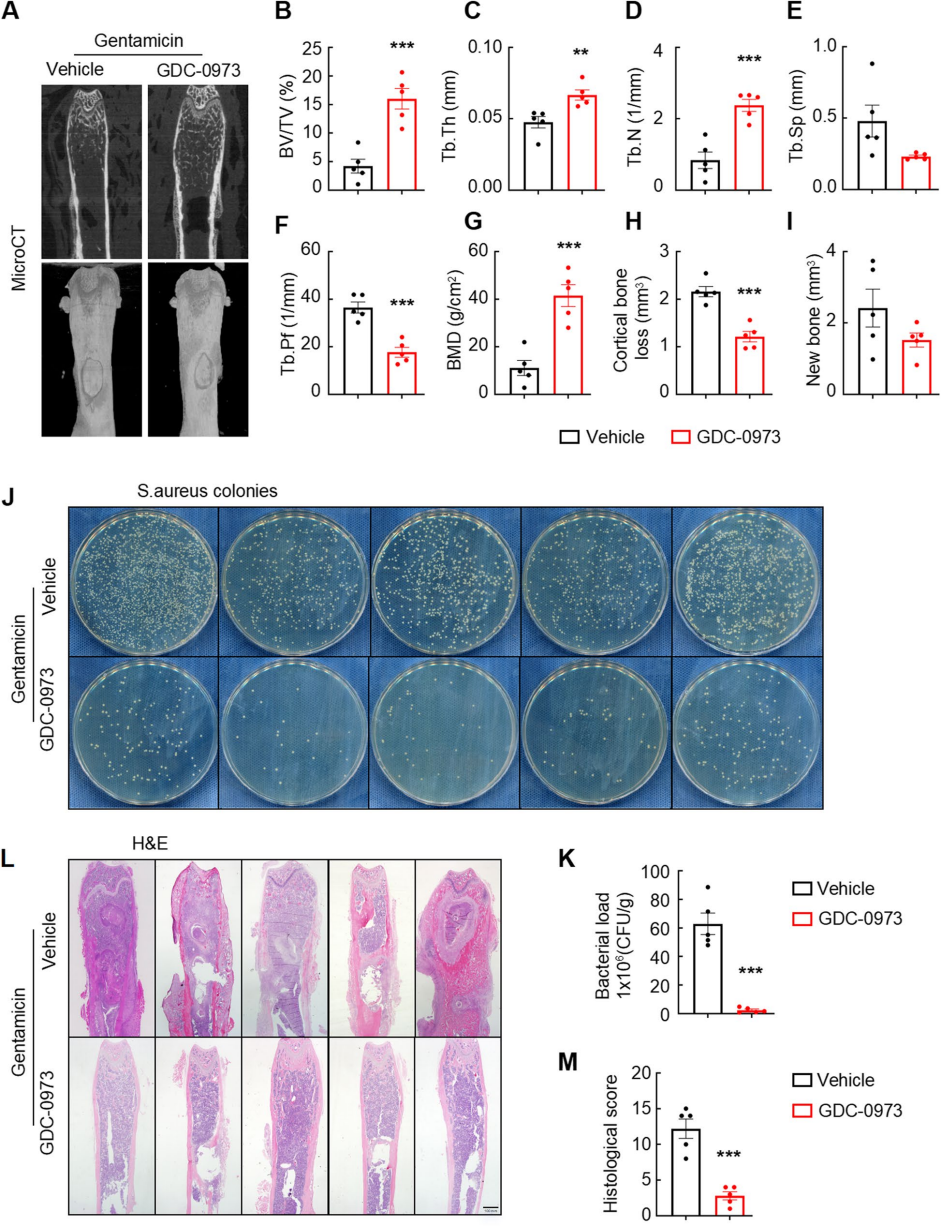
The inhibition of MEK1 signaling attenuates *S. aureus*-induced osteomyelitis by reducing bone destruction and the bacterial burden. **A**–**I** Representative coronal micro-CT images (**A**) and quantitative analysis of the bone volume/tissue volume (**B**), trabecular thickness (**C**), trabecular number (**D**), trabecular separation (**E**), trabecular bone pattern factor (**F**), bone mineral density (**G**), cortical bone loss (**H**) and new bone formation (**I**) of the femora from mice with *S. aureus*-induced osteomyelitis treated with the MEK1 inhibitor GDC-0973. **J** and **K** Representative images (**J**) and quantitative (**K**) analysis of the bacterial load in the *S. aureus*-infected femurs of mice treated with the MEK1 inhibitor GDC-0973 compared with those treated with the vehicle. (L and M) Representative images (**L**) and quantitative (**M**) analysis of H&E-stained *S. aureus*-infected femurs from mice treated with GDC-0973 compared with those treated with the vehicle. Scale bars, 100 μm. *n* = 5 mice/group, *p < 0.05, **p < 0.01, and ***p < 0.001

We investigated the role of MEK1 signaling in modulating *S. aureus*-induced osteomyelitis pathogenesis by establishing a murine femoral infection model through *S. aureus* inoculation and subsequently treated the animals with combination therapy comprising GDC-0973 (a selective MEK1 inhibitor) and gentamicin. The micro-CT analysis of femurs harvested on Day 14 postinfection revealed distinct pathological features between the vehicle-treated and GDC-0973-treated groups. Vehicle-treated mice presented pronounced cortical bone loss and peri-implant reactive bone formation along the intramedullary implant path. In contrast, compared with vehicle-treated mice, GDC-0973-treated mice presented a marked preservation of cancellous bone architecture along with reduced osteolytic damage (Fig. 3A). The quantitative trabecular microstructure analysis further corroborated these findings. GDC-0973 treatment significantly rescued the bone volume/tissue volume (BV/TV) in the distal femur, driven primarily by improvements in the trabecular thickness (Tb.Th) and trabecular number (Tb.N) (Fig. 3B and D). Additionally, the trabecular bone pattern factor (Tb.Pf), a marker of structural connectivity, was substantially increased in the treatment group (Fig. 3F). However, trabecular separation (Tb.Sp) remained unchanged between the cohorts (Fig. 3E). Consistent with the observed structural improvements, the bone mineral density (BMD) of GDC-0973-treated mice was significantly greater than that of vehicle control-treated mice (Fig. 3G). The cortical bone analysis revealed a statistically significant reduction in cortical bone loss with MEK1 inhibition, whereas reactive bone formation showed a modest, nonsignificant trend toward improvement (Fig. 3H and I).

Given that the blockade of MEK1 signaling can significantly ameliorate cortical bone loss and trabecular bone destruction, we hypothesized that inhibiting MEK1 signaling might also diminish the bacterial load. In support of this hypothesis, bacterial plates were coated with infected bone tissues, and GDC-0973-treated mice presented a notable reduction in bacterial colony-forming units (CFUs) per gram compared with vehicle-treated mice via the classical culture-based analysis (Fig. 3J and K). Furthermore, H&E staining of infected femoral tissues further showed that blocking MEK1 signaling significantly mitigated bone destruction. Consistent with the micro-CT imaging findings, mice treated with GDC-0973 presented an improved cancellous bone mineral density and quality. In contrast, vehicle-treated mice displayed substantial inflammatory cell infiltration in the peri-implant bone marrow, abscess formation in the intramedullary and metaphyseal regions, and extensive reactive new bone formation around the cortical bone (Fig. 3L). Blinded histological scoring of bone sections revealed the significant amelioration of bone destruction in GDC-0973-treated mice compared with vehicle-treated mice (Fig. 3M). Taken together, these results suggest that the overactivation of MEK1 signaling is a critical factor contributing to the reduced bacterial immune defenses.

### Blockade of the MEK1-ERK1/2 cascade in macrophages leads to elevated mtROS levels and enhanced bactericidal efficacy

**Fig. 4:**
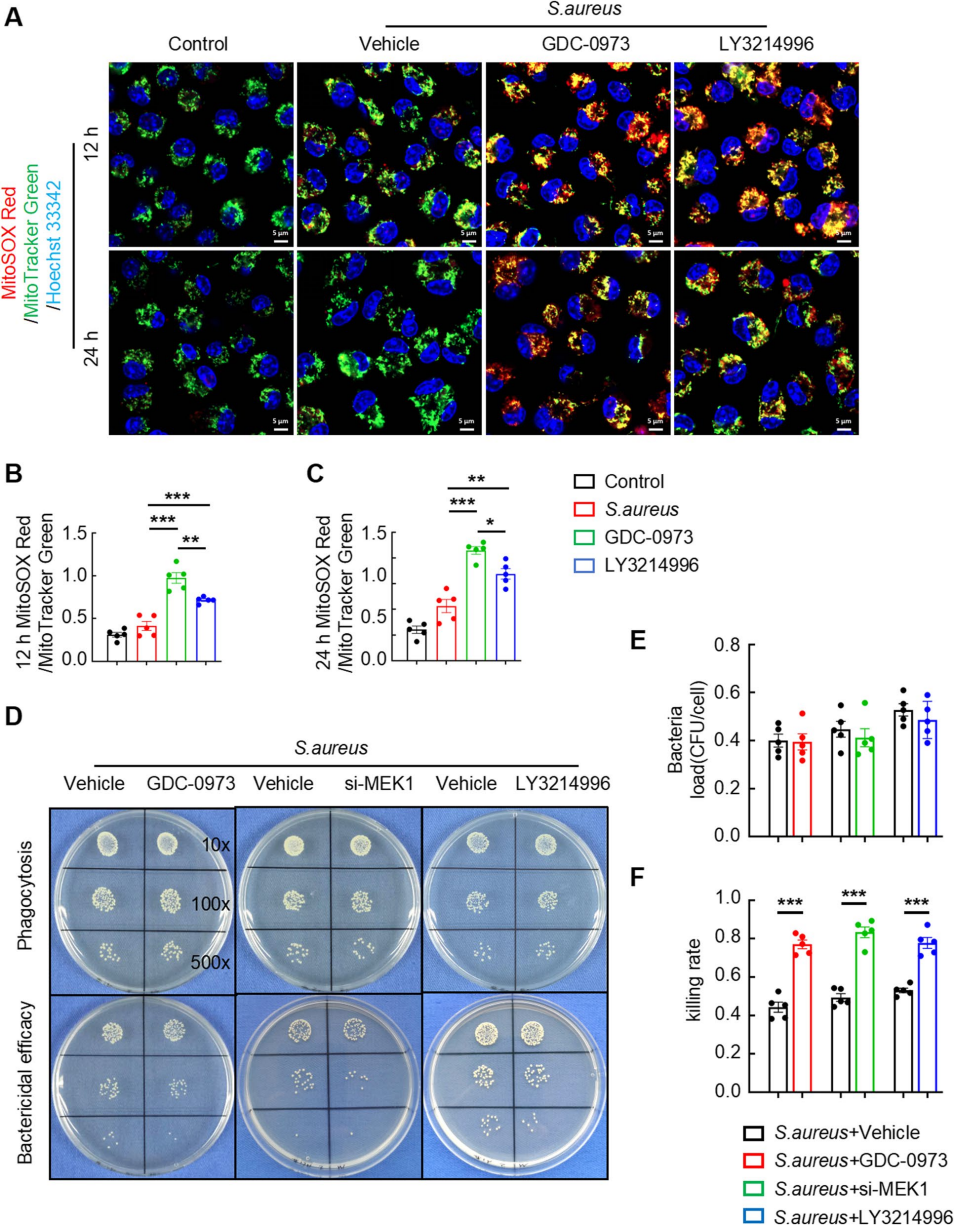
Blockade of the MEK1-ERK1/2 cascade in macrophages results in elevated mtROS levels and enhanced bactericidal efficacy. **A**–**C** Representative images (**A**) and quantification (**B** and **C**) of mtROS levels in *S. aureus*-infected BMDMs treated with or without GDC-0973 or LY3214996 for 12 and 24 h. Scale bars, 5 μm. **D**–**F** Representative images (**D**) and quantification (**E **and **F**) of colony-forming units (CFUs) of *S. aureus* from the phagocytosis (1 h) and bactericidal (12 h) assays. BMDMs were pretreated with the vehicle, GDC-0973, si-MEK1 or LY3214996 and infected with *S. aureus* (MOI = 10). *n* = 5 samples/group, *p < 0.05, **p < 0.01, and ***p < 0.001

Mounting evidence has established mtROS as essential mediators of the bactericidal activity of macrophages (Li et al. 2023; Jin et al. 2024; Fernandez-Boyanapalli et al. 2015). Based on this bactericidal paradigm, we postulated that the MEK1-ERK1/2 signaling axis exerts negative regulatory control over mtROS production. We investigated the levels of mtROS, presented as the ratio of MitoSOX Red to MitoTracker Green, in BMDMs stimulated with *S. aureus* at various time points via immunofluorescence microscopy (IFM) to delineate the bactericidal feedback mechanisms mediated by mtROS in macrophages. As revealed by the data, the biphasic mtROS response in *S. aureus*-challenged macrophages peaked at 4 h postinfection, followed by progressive attenuation up to 24 h (Figure S1 C and S1D). Pharmacological intervention with either the MEK1 inhibitor GDC-0973 or the ERK1/2 inhibitor LY3214996 reinstated mtROS generation at later infection stages (12 h/24 h), overcoming pathogen-induced suppression (Fig. 4A). Comparative quantification revealed that both inhibitors significantly increased mtROS production relative to that of the vehicle controls at 12 h and 24 h (Fig. 4B and C). Notably, MEK1 inhibition elicited greater mtROS potentiation than direct ERK1/2 blockade, consistent with the hierarchical position of MEK1 upstream of ERK1/2 in the signaling cascade.

Given the established correlation between mtROS generation and the bactericidal efficacy of macrophages against *S. aureus*, we investigated whether *S. aureus*-induced hyperactivation of the MEK1-ERK1/2 axis functionally compromises antimicrobial responses. The classical culture-based analysis revealed that the phagocytic ability of GDC-0973-treated, MEK1-silenced or LY3214996-treated BMDMs was comparable to that of vehicle-treated BMDMs (Fig. 4D and E), indicating preserved pathogen internalization, irrespective of pathway inhibition. Strikingly, MEK1-ERK1/2 blockade profoundly enhanced intracellular bacterial clearance (Fig. 4D and F). This dissociation between intact phagocytosis and enhanced bactericidal activity implicates MEK1-ERK1/2 signaling specifically in postinternalization microbial killing. Collectively, our findings confirm that *S. aureus* inhibits the bactericidal efficacy of macrophages through pathogen-induced hyperactivation of the MEK1-ERK1/2 signaling axis and repressing mtROS generation.

### MEK1-ERK1/2 pathway activation suppresses MtROS production by enhancing macrophage mitophagy

**Fig. 5:**
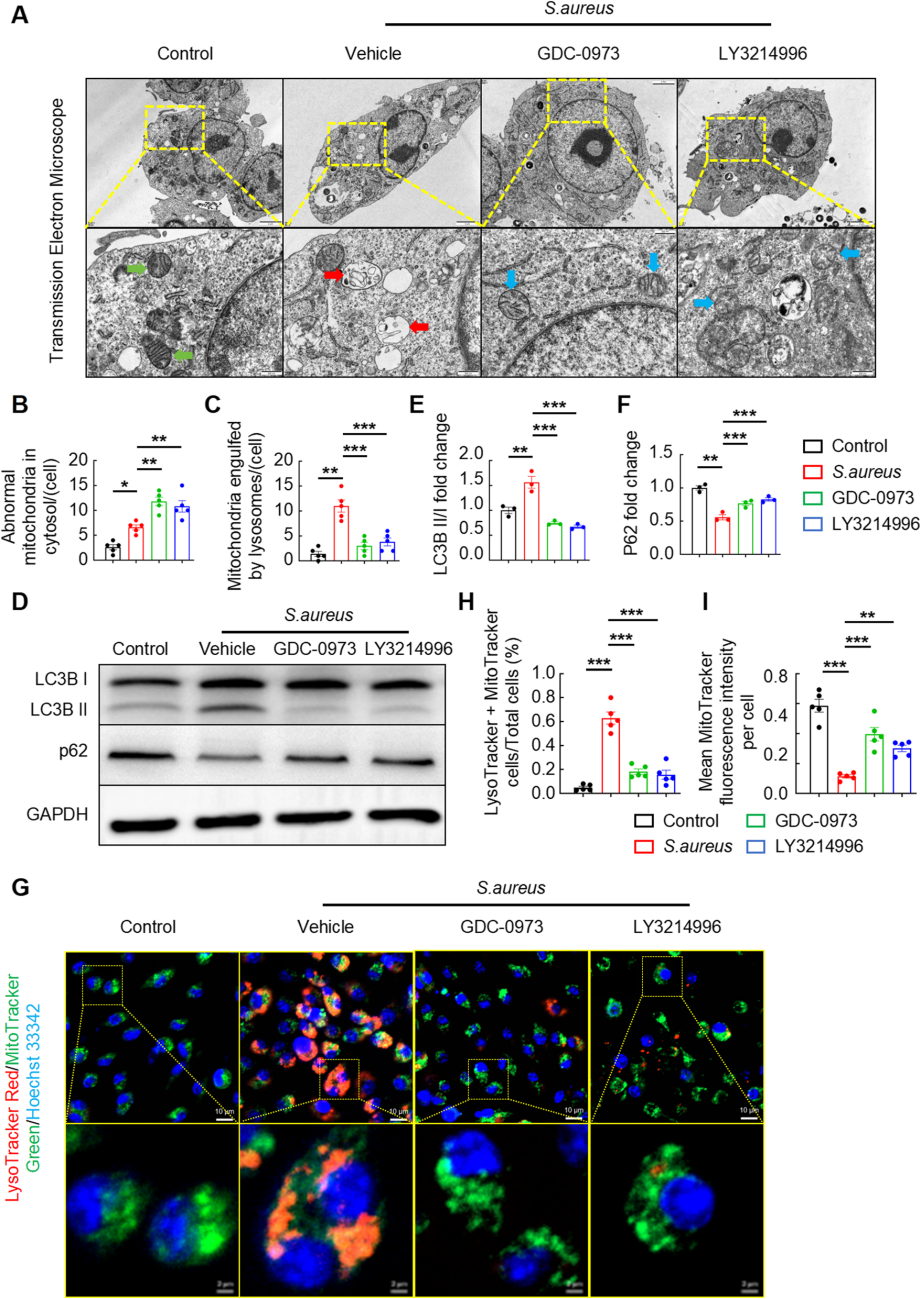
MEK1-ERK1/2 pathway activation suppresses mtROS production by enhancing mitophagy in macrophages. **A**–**C** Representative images (**A**) and quantification (**B** and **C**) of TEM images of BMDMs infected with *S. aureus* (MOI = 10) for 12 h and treated with or without GDC-0973 or LY3214996. Normal mitochondria (green arrows), dissolved mitochondria (red arrows), and abnormal mitochondria (blue arrows) are shown. Scale bars, 2 μm, 500 nm; *n* = 5 samples/group. **D**–**F** Representative images (**D**) and quantification (**E** and **F**) of the western blot results for the LC3B and p62 protein levels in BMDMs infected with *S. aureus* (MOI = 10) for 12 h and treated with or without GDC-0973 or LY3214996. *n* = 3 samples/group. **G**–**I** Representative images (**G**) and quantification (**H** and **I**) of LysoTracker/MitoTracker staining in BMDMs infected with *S. aureus* (MOI = 10) for 12 h and treated with or without GDC-0973 or LY3214996. Scale bars, 10 μm; *n* = 5 samples/group. *p < 0.05, **p < 0.01, and ***p < 0.001

Emerging evidence indicates that mitophagy in macrophages is increased under pathological conditions, resulting in reduced mtROS release (Su et al. 2023). Building on this paradigm, we postulate that hyperactivation of the MEK1-ERK1/2 signaling axis may attenuate mtROS generation through the potentiation of macrophage mitophagy during *S. aureus* infection. Mechanistically, mitophagy involves the selective encapsulation of damaged mitochondria within autophagosomes to generate mitophagosomes, which subsequently fuse with lysosomes for terminal degradation via lysosomal hydrolases (Mijaljica et al. 2007). Our transmission electron microscopy (TEM) analysis revealed that *S. aureus*-stimulated BMDMs underwent mitophagy, characterized by lysosomal‒mitochondrial fusion and subsequent hydrolytic degradation (indicated by red arrows). However, treatment with GDC-0973 or LY3214996 abolished this mitochondrial–lysosomal fusion (Fig. 5A). The quantitative analysis revealed a significant increase in lysosome-engulfed mitochondria in the infection group compared with the untreated control group, whereas inhibitor treatment markedly reduced this phenomenon (Fig. 5C). Intriguingly, compared with the control groups, the infected and inhibitor-treated groups presented mitochondrial structural abnormalities. Specifically, inhibitor-treated cells displayed a disruption of mitochondrial cristae (blue arrows), whereas control mitochondria maintained an intact architecture (green arrows). Notably, the number of structurally abnormal mitochondria in the cytosol was significantly greater in the inhibitor groups than in the infection groups (Fig. 5B). These findings suggested that treatment with GDC-0973 or LY3214996 could rescue mitochondria that were compromised by *S. aureus*.

We further confirmed that the blockade of the MEK1-ERK1/2 pathway suppresses *S. aureus*-induced mitophagy in macrophages by performing western blot analyses of LC3B-I/II conversion and p62 and GAPDH protein levels in GDC-0973- or LY3214996-treated BMDMs. During mitochondrial autophagy, the LC3-I protein is modified to form the LC3-II protein, promoting the fusion of autophagosomes with lysosomes and encapsulating p62, which is a selective autophagy receptor and plays an important role in mitophagy by recognizing and binding to ubiquitinated mitochondrial proteins, thereby completing the degradation of damaged mitochondria (Xu et al. 2020). Consistent with this mechanism, immunoblotting revealed that *S. aureus* infection significantly increased the LC3B-II/I ratio while reducing p62 levels. However, pharmacological inhibition with GDC-0973 or LY3214996 reversed these changes (Fig. 5D). Compared with the blank controls, the infection group presented a marked increase in the LC3B-II/I ratio, indicating increased autophagosome formation. Notably, this increase was effectively suppressed following inhibitor administration (Fig. 5E). Conversely, infection significantly depleted p62 protein levels, suggesting accelerated autophagic degradation. This pathological decrease was markedly reversed by the inhibitor treatment (Fig. 5F). Furthermore, the LysoTracker Red/MitoTracker Green was determined via IFM to investigate mitophagy in *S. aureus*-infected BMDMs. The results revealed a remarkable increase in the fusion of lysosomes and mitochondria in the vehicle-treated group compared with the control-treated group, whereas the GDC-0973-treated and LY3214996-treated groups presented a notable reduction compared with the vehicle-treated group (Fig. 5G). Consistent with these findings, the LysoTracker Red and MitoTracker Green colocalization in the vehicle-treated group increased markedly relative to that in the other three groups (Fig. 5H). Correspondingly, the mean MitoTracker Green fluorescence intensity was significantly decreased in the vehicle-treated group compared with the other three groups (Fig. 5I).Taken together, the persistent activation of the MEK1-ERK1/2 cascade signaling pathways evoked by *S. aureus* suppressed mtROS production through an increase in mitophagy in macrophages.

### MEK1-ERK1/2 activation enhances mitophagy by inhibiting CHEK2 expression to reduce MtROS levels

**Fig. 6:**
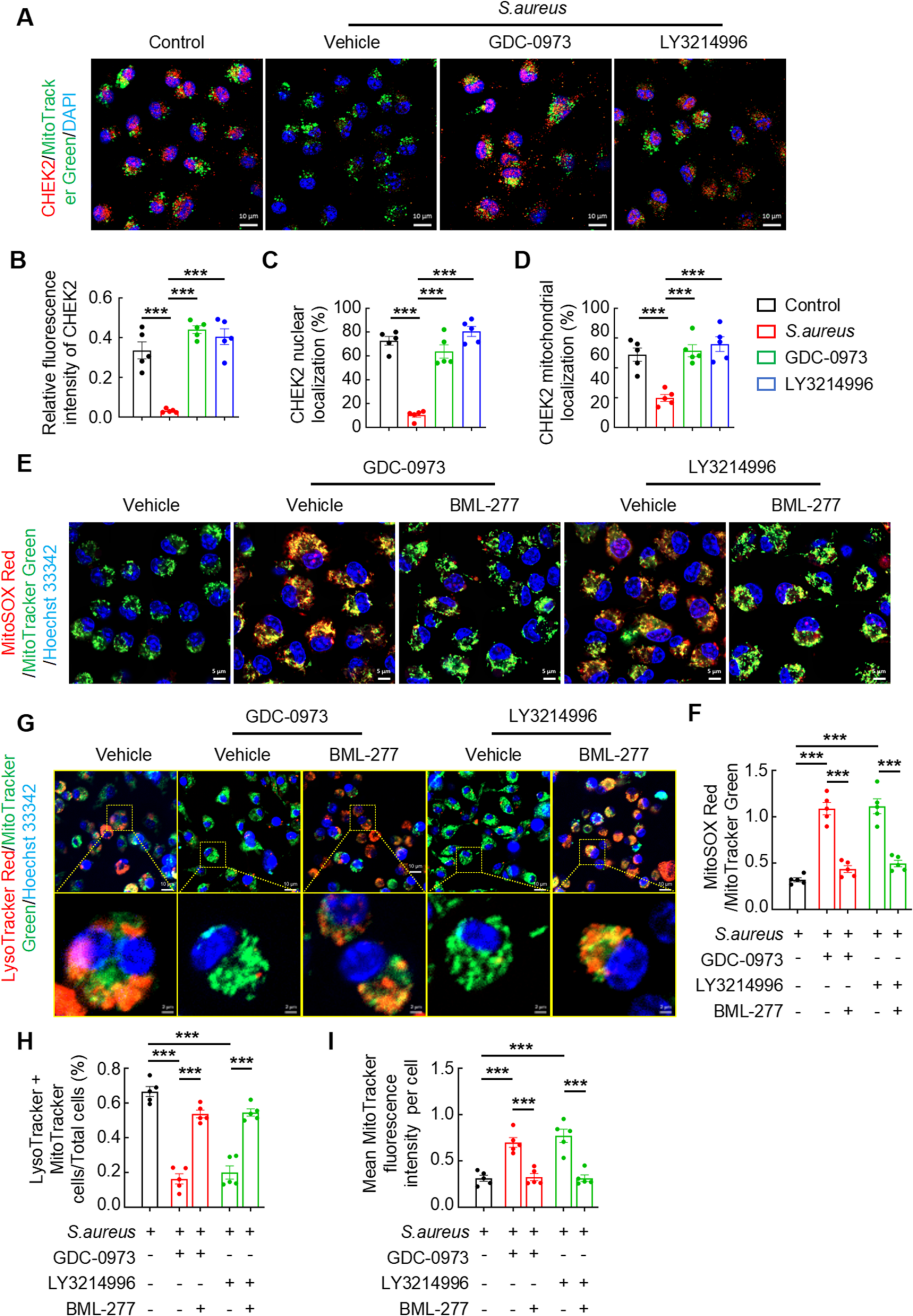
MEK1-ERK1/2 activation in macrophages enhances mitophagy by inhibiting CHEK2 expression to reduce mtROS levels. **A**–**D** Representative images (**A**) and quantification (**B**–**D**) of CHEK2 levels in BMDMs infected with *S. aureus* (MOI = 10) for 12 h and treated with or without GDC-0973 or LY3214996. Statistical results of CHEK2 fluorescence intensity (**B**), mitochondrial localization of CHEK2 (**C**), and nuclear localization of CHEK2 (**D**). Scale bars, 10 μm. **E** and **F** Representative images (**E**) and quantification (**F**) of mtROS levels in BMDMs infected with *S. aureus* for 12 h and treated with GDC-0973 or LY3214996 and BML-277. Scale bars, 5 μm. **G**–**I** Representative images (**G**) and quantification (**H** and **I**) of LysoTracker/MitoTracker staining in BMDMs infected with *S. aureus* (MOI = 10) for 12 h and treated with GDC-0973 or LY3214996 and BML-277. Scale bars, 10 μm. *n* = 5 samples/group, ***p < 0.001

Identifying the downstream signaling mediators linking the MEK1-ERK1/2 and mitophagy pathways is critical to obtain deeper insights into how MEK1-ERK1/2 activation mediates mitophagy. This investigation was further motivated by our published findings showing that *S. aureus*-induced mitochondrial damage in macrophages can be functionally rescued through CHEK2 translocation to mitochondria (Jin et al. 2024). Cell cycle checkpoint kinase 2 (Chek2) is a key regulatory gene in cell cycle control, essential for activating the DNA damage response. Based on our mechanistic framework, we postulated that CHEK2 serves as the critical signaling intermediary connecting MEK1-ERK1/2 activation to mitophagy regulation. We initially evaluated the colocalization of CHEK2 with red fluorescence and mitochondria with MitoTracker Green fluorescence in *S. aureus*-infected BMDMs to validate this hypothesis. As hypothesized, *S. aureus* infection markedly suppressed CHEK2 expression in BMDMs, whereas pharmacological inhibition with GDC-0973 or LY3214996 effectively restored CHEK2 levels (Fig. 6A). Fluorescence quantification further revealed a pronounced reduction in the CHEK2 intensity in vehicle-treated infected cells compared with that in uninfected controls. In contrast, both the MEK1 and ERK1/2 inhibitor-treated groups presented significant CHEK2 upregulation compared with the vehicle-treated infected group (Fig. 6B). Crucially, this restored CHEK2 expression correlated with its subcellular redistribution—shifting from nuclear retention following infection to mitochondrial translocation upon kinase inhibition. Consistent with this mechanistic shift, quantitative imaging revealed a substantial increase in the mitochondrial-to-nuclear CHEK2 localization ratio in inhibitor-treated *S. aureus*-infected BMDMs (Fig. 6C and D).

To date, the potential involvement of CHEK2 in mtROS modulation remains undefined. We addressed this gap by investigating the functional relationship between CHEK2 activity and mtROS generation in *S. aureus*-infected BMDMs using IFM. Strikingly, pharmacological inhibition of GDC-0973 or LY3214996 in infected cells potentiated mtROS production. Conversely, cotreatment with the CHEK2 inhibitor BML-277 and either the GDC-0973 or LY3214996 inhibitor reversed this increase in mtROS levels (Fig. 6E). A quantitative analysis of the MitoSOX Red/MitoTracker Green fluorescence ratios confirmed significant increases in mtROS levels in the GDC-0973- or LY3214996-treated groups compared with the vehicle-treated controls. In contrast, the dual inhibition of MEK1/ERK1/2 and CHEK2 (GDC-0973 + BML-277 or LY3214996 + BML-277) markedly reduced the mtROS ratio compared with that in the monotherapy groups (Fig. 6F). Correspondingly, the mitophagy detection results aligned with the aforementioned trend. The LysoTracker Red/MitoTracker Green colocalization analysis indicated that pharmacological inhibition with GDC-097 or LY3214996 in *S. aureus*-infected BMDMs significantly suppressed mitophagy. Conversely, combining BML-277 with either compound enhanced mitophagy (Fig. 6G). The quantitative analysis confirmed a substantial reduction in mitophagy levels in the GDC-0973- or LY3214996-treated groups compared with the vehicle-treated controls. Compared with monotherapy, dual inhibition with GDC-0973 + BML-277 or LY3214996 + BML-277 significantly increased the LysoTracker Red and MitoTracker Green colocalization (Fig. 6H). However, the mean MitoTracker Green fluorescence intensity displayed an inverse trend, which can be attributed to mitochondrial-lysosomal fusion events following mitophagy induction (Fig. 6I). Furthermore, to mechanistically delineate the role of mitophagy in sustaining intracellular bacterial survival within macrophages, we quantitatively assessed the phagocytic and killing efficiency of BMDMs following pharmacological autophagy inhibition (Mdivi-1). The classical culture-based analysis revealed that the phagocytic ability of Mdivi-1-treated was comparable to that of vehicle-treated BMDMs (Figure S1E and S1G). While autophagy blockade profoundly enhanced intracellular bacterial clearance (Figure S1 F and S1H). These data indicate that *S. aureus* infection in macrophages triggers the MEK1-ERK1/2-CHEK2 signaling axis to regulate pathogen-induced mitophagy. Pharmacological inhibition of MEK1-ERK1/2 signaling significantly increases CHEK2 expression, thereby suppressing mitophagy and subsequently increasing mtROS generation.

### Mitophagy in macrophages regulated by the MEK1-ERK1/2-CHEK2 signaling pathway is crucial for the progression of *S. aureus*-induced osteomyelitis

**Fig. 7:**
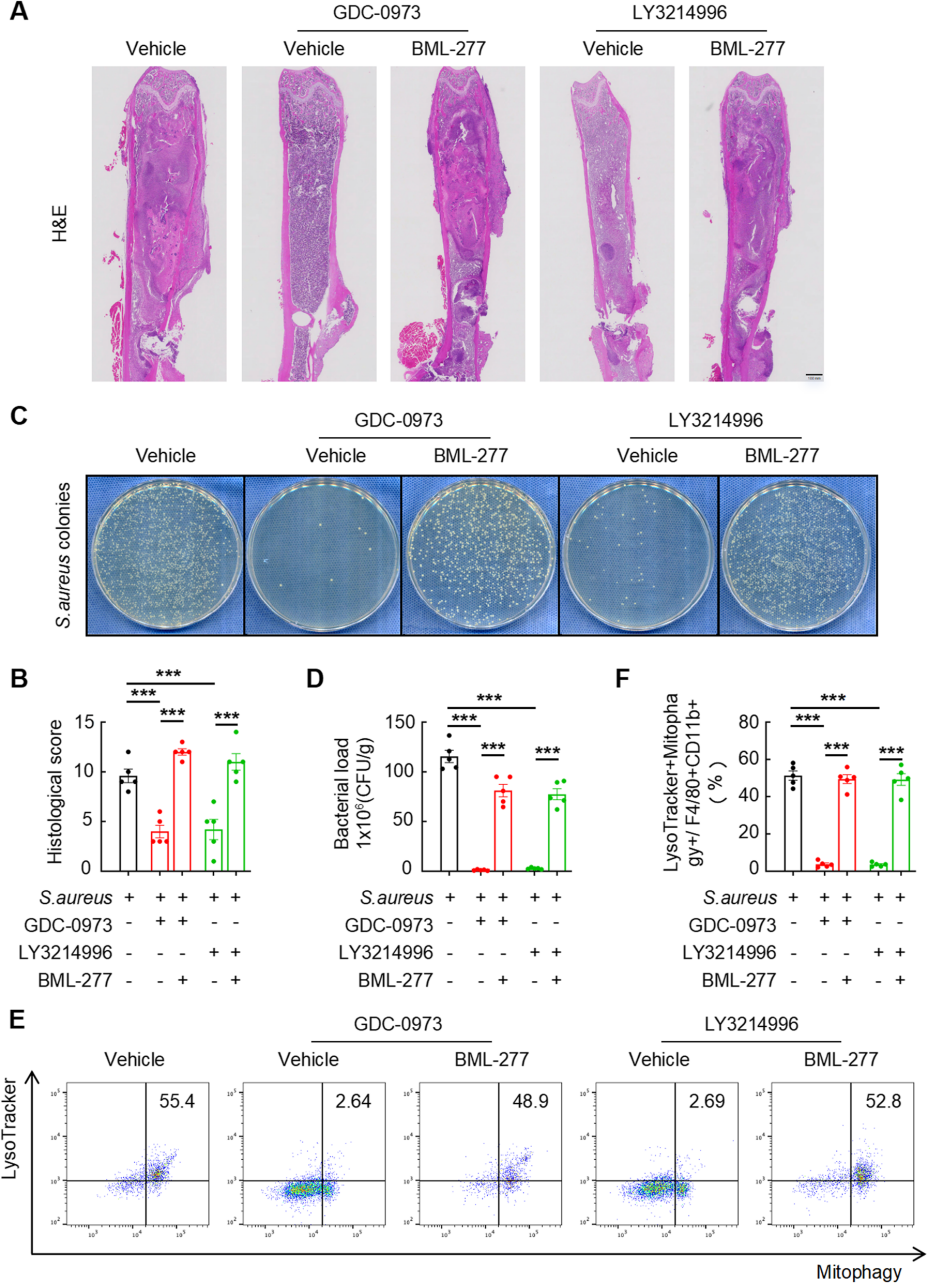
Mitophagy in macrophages regulated by the MEK1-ERK1/2-CHEK2 signaling pathway is crucial for the progression of *S. aureus*-induced osteomyelitis. **A** and **B** Representative images (**A**) and quantitative (**B**) analysis of H&E staining of *S. aureus*-infected femurs from mice treated with or without GDC-0973 or LY3214996 and BML-277 compared with those treated with the vehicle. Scale bars, 100 μm. **C** and **D** Representative images (**C**) and quantitative (**D**) analysis of the bacterial load in the *S. aureus*-infected femurs of mice treated with or without GDC-0973 or LY3214996 and BML-277 compared with those treated with the vehicle. **E** and **F** Representative images (**E**) and quantification (**F**) of the flow cytometry results for the proportions of LysoTracker + Mitophagy+/F4/80 + CD11b + cells in the *S. aureus*-infected femurs of mice treated with or without GDC-0973 or LY3214996 and BML-277 compared with those treated with the vehicle. *n* = 5 mice/group, ***p < 0.001

We previously showed that the MEK1-ERK1/2-CHEK2 signaling pathway diminishes mtROS release in macrophages by increasing mitophagy in vitro. A mouse model of *S. aureus* infection implanted in the femur for 14 days was treated with different combinations of inhibitors to validate this mechanism in vivo. As anticipated, the results of H&E staining of the infected femurs after GDC-0973 or LY3214996 treatment clearly revealed the alleviation of cancellous bone destruction and reactive hyperplasia of cortical bone. Importantly, combination therapy with BML-277 paradoxically reversed these protective effects, with the GDC-0973 + BML-277 and LY3214996 + BML-277 groups exhibiting exacerbated bone destruction and abscess formation compared with the monotherapy groups (Fig. 7A). Blinded histological scoring confirmed this pattern: the scores of the combination therapy group not only showed no improvement compared with the vehicle controls but were also significantly higher (indicating worse pathology) than monotherapy groups (Fig. 7B). Moreover, bacterial quantification revealed similar patterns: combination therapies (GDC-0973 + BML-277 or LY3214996 + BML-277) resulted in significantly greater bacterial loads than did the corresponding monotherapies. No changes in bacterial CFUs per gram were observed using the classical culture-based analysis compared with the vehicle-treated group (Fig. 7C and D).

Taken together, our data indicate that MEK1-ERK1/2 inhibition ameliorates *S. aureus*-induced osteolysis and bacterial persistence via the upregulation of CHEK2 expression. As these effects are independent of mitophagy, we subsequently investigated the correlation between mitophagy and the MEK1-ERK1/2-CHEK2 axis in vivo via flow cytometry. The results of the LysoTracker^+^Mitophagy^+^*/*F4/80^+^CD11b^+^ ratio revealed significantly lower mitophagy levels in the monotherapy groups (GDC-0973 or LY3214996) than in the vehicle-treated controls, whereas the combination therapy groups (GDC-0973 + BML-277 or LY3214996 + BML-277) exhibited markedly greater mitophagy activity (Fig. 7E and F). Taken together, our findings establish the MEK1-ERK1/2-CHEK2 axis as a critical regulator of mitophagy in *S. aureus*-induced osteomyelitis. Mechanistically, pathogen-activated MEK1-ERK1/2 signaling in macrophages drives osteolytic progression through CHEK2 suppression, which coordinates reduced mtROS release via increased mitophagy, ultimately compromising bacterial clearance. Pharmacological inhibition reverses this pathological cascade: restored CHEK2 expression attenuates mitophagy, amplifies mtROS-mediated bactericidal activity, and concomitantly reduces both the microbial burden and bone destruction.

## Discussion

The management of *Staphylococcus aureus*-induced osteomyelitis remains clinically intractable because of the pathogen’s sophisticated pathoadaptive manipulation of the skeletal immunometabolic niche (Sweet et al. 2025). Our investigation revealed that the macrophage MEK1 signaling axis is selectively activated during *S. aureus* invasion, which mechanistically governs antimicrobial effector functions through a previously unrecognized CHEK2-mitophagy regulatory cascade. These findings establish novel theoretical foundations for targeting the osteoimmune microenvironment in the treatment of osteomyelitis.

Unlike conventional broad-spectrum MEK1/2 inhibitors, we show that MEK1 activation specifically triggers ERK1/2 signaling in *S. aureus*-challenged macrophages, whereas MEK2 signaling homeostasis remains unaffected. This specificity challenges the established view of the roles of MEK1/2 in antibacterial immunity, revealing functional compensation within MEK1/2 signaling and demonstrating that selective targeting of MEK1 may improve therapeutic specificity while minimizing side effects. The experimental evidence indicates that dual Mek1/2 knockdown eliminates Erk1/2 phosphorylation, triggering reduced proliferation, apoptotic cell death, cutaneous barrier impairment, and lethality. In contrast, the retention of a single functional allele from either gene maintains normal developmental processes (Scholl et al. 2007). These observations confirm that Mek1/2 are functionally redundant and function as a linear signaling node in the MAPK cascade to regulate mammalian development and tissue homeostasis. Furthermore, in cancer treatment, nonselective MEK1/2 inhibitors cause more adverse effects than do selective MEK1 or MEK2 inhibitors (Caunt et al. 2015). Collectively, these findings indirectly underscore the importance of our research in exploring safer and more effective therapeutic approaches for treating osteomyelitis.

Intriguingly, our findings reveal a dynamic shift in macrophage behavior during *S. aureus* infection: the p-MEK1^+^ F4/80^+^ macrophage subpopulation expands prominently in the early-to-mid-infection phases but decreases substantially at later stages. We attribute this phenomenon to macrophage depletion, a pathological state driven by the multifaceted immunosuppressive strategies of *S. aureus*. The pathogen subverts macrophage functionality through toxin release, metabolic sabotage, and the exploitation of immune checkpoints (Arciola et al. 2018; Miller LS et al. 2020). For example, biofilm-associated virulence factors trigger NLRP3 inflammasome activation in macrophages, promoting pyroptosis and subsequently triggering this critical immune cell population (Wang X et al. 2022). Therapeutically, PD-1 checkpoint blockade not only rejuvenates exhausted macrophages but also restores their bactericidal efficacy, ultimately reducing osteomyelitic bone damage (Li et al. 2023). Mirroring this therapeutic promise, our animal osteomyelitis model showed that MEK1 inhibition markedly attenuated bone destruction scores, underscoring its dual mechanism of action—simultaneously curbing bacterial persistence and preserving bone integrity. We propose that MEK1 signaling blockade may counteract macrophage exhaustion by revitalizing their antimicrobial functions, thereby interrupting the vicious cycle of infection-driven osteolysis.

From a mechanistic perspective, our work expands the theoretical framework of the regulation of mitochondrial dynamics in innate immunity. While previous studies have focused primarily on the m-TOR or AMPK pathways in mitophagy regulation (Herzig S et al. 2018; Chen Q et al. 2022; Yau WW et al. 2018), we established the MEK1‒CHEK2 axis as a novel regulator of mitochondrial quality control, providing molecular evidence for the “pathogen‒mitochondria interaction” theory. Genetic and pharmacological inhibition of MEK1 attenuated mitophagy via CHEK2-dependent mechanisms, reconciling the observation of elevated mtROS levels alongside improved bacterial clearance. A “threshold effect” model has been proposed (Mijaljica et al. 2007): moderate mitophagy sustains metabolic homeostasis, whereas excessive mitophagy depletes functional mitochondria, attenuating ROS production—a concept that aligns with the biphasic role of mitophagy in sepsis (Patoli D et al. 2020). Moreover, since the pioneering work of Kim et al. (Kim et al. 2014) first revealed that pathogens suppress host immune responses by hijacking mitochondrial quality control mechanisms, subsequent studies have corroborated that *S. aureus* evades immunity through mitophagy induction in macrophages, thereby inhibiting mtROS production (Yang et al. 2025; Yang B et al. 2024; Wang et al. 2023). Therapeutic interventions targeting key pathways—such as PD-L1/PD-1 blockade (Li et al. 2023), PINK1/Parkin inhibition (Zhou et al. 2023), or NIX/BNIP3 suppression (Clague MJ et al. 2025)—have been shown to significantly attenuate mitophagy, restore mtROS levels, and enhance host defenses. Our study adds a novel layer to this mechanistic framework by identifying the MEK1-CHEK2 axis as a critical regulator of mitochondrial homeostasis and macrophage function during *S. aureus* infection.

CHEK2, a critical regulator of cell cycle progression and the DNA damage response (DDR) (Xu P et al. 2024), has emerged as a central player in a novel MEK1-CHEK2 signaling axis that links the DDR with mitochondrial quality control. Our findings indicate that pharmacological or genetic inhibition of the MEK1-ERK1/2 pathway triggers CHEK2 upregulation, followed by its nuclear-to-mitochondrial translocation to facilitate the repair of damaged mitochondrial function, which in turn suppresses mitophagy. Based on our findings, we propose that CHEK2 translocation to mitochondria could specifically orchestrate the repair machinery for mitochondrial DNA (mtDNA) damage, although this hypothesis requires rigorous experimental validation. Notably, although CHEK2 is canonically activated downstream of ATM/ATR kinases during genotoxic stress (Khashab F et al. 2021), our previous work revealed its noncanonical role as a downstream target of MEK1/2 signaling involved in the regulation of mitochondrial ROS release (Jin et al. 2024), a mechanism that deserves further investigation. Studies have shown that under conditions such as DNA damage or energy stress, the ATM-CHEK2 axis activates autophagy to maintain ROS homeostasis in response to oxidative stress (Shen et al. 2019; Guo QQ et al. 2020). Additionally, CHEK2 phosphorylates p53 to increase its interaction with Parkin, facilitating the ubiquitination and autophagic clearance of damaged mitochondria (Agborbesong E et al. 2023; Huang et al. 2025). While the regulatory nexus between CHEK2 and autophagic responses during *S. aureus* infection remains enigmatic, our study provides groundbreaking mechanistic insights into this underexplored biological interface. Through systematic interrogation, we established that pathogenic challenge by *S. aureus* activates the MEK1-ERK1/2 signaling cascade in macrophages, enhances mitophagy through CHEK2 suppression, and decreases mtROS generation, ultimately compromising antimicrobial effector functions. Crucially, although these findings position CHEK2 as a MEK1-regulated mitophagy inhibitor, the molecular mechanisms underlying CHEK2-mediated repair of mitochondrial function remain unresolved. Further exploration of how mitochondrial surveillance pathways are activated during infection has profound implications for the treatment of osteomyelitis.

Clinically, our findings provide clinically actionable insights for the development of tissue-selective MEK1 inhibitors to minimize systemic toxicity while preserving therapeutic efficacy. Furthermore, our in vivo experiments employed MEK1 inhibitor dosages at one-tenth of the therapeutic level used in oncology and showed significantly reduced adverse effect profiles. However, in vitro dose‒response analyses have revealed a critical challenge: MEK1 inhibition has a narrow therapeutic window where effective bactericidal activity overlaps with the cytotoxic thresholds in macrophages. This pharmacological constraint necessitates the development of nanoparticle-enabled delivery platforms to increase drug accumulation at disease sites while sparing healthy tissues (Tang et al. 2024; Li Z et al. 2023)—a strategy that could simultaneously optimize pharmacokinetic profiles and mitigate off-target liabilities.

This study has inherent limitations: (1) mechanistic validation remains based on cellular models, necessitating macrophage-specific MEK1-knockdown models for in vivo confirmation; (2) the downstream effectors of CHEK2 in mitophagy regulation require elucidation; and (3) murine data need validation in clinical samples (e.g., MEK1 activity in CD68^+^ macrophages from osteomyelitis patients). Future work should (1) decipher the spatial regulatory mechanisms of the MEK1-CHEK2 signaling axis (e.g., mitochondrial–nuclear shuttling) and (2) explore the universality of this regulatory pathway in other intracellular bacterial infections.

## Conclusions

This study delineates MEK1 signaling in macrophages as a central regulator of *S. aureus*-induced osteomyelitis, orchestrating mitophagy and host defenses through the epigenetic control of CHEK2. These discoveries not only deepen the understanding of the bone immunometabolic microenvironments but also provide direct theoretical evidence for the development of selective MEK1 inhibitors. Future research may leverage this pathway to design macrophage-targeted therapeutics, providing potential breakthroughs in overcoming current therapeutic bottlenecks for osteomyelitis.

## Supplementary Information

Supplementary Material 1.

## Data Availability

No datasets were generated or analysed during the current study.

## Abbreviations

*S.* *aureus*: *Staphylococcus aureus*
mtROS: Mitochondrial reactive oxygen species
BMDMs: Bone marrow-derived macrophages
TSA: Tryptic soy agar
TSB: Tryptic soy broth
SPF: Specific pathogen-free
PBS: Phosphate-buffered saline
BMD: Bone mineral density
H&E: Hematoxylin and eosin
EDTA: Ethylenediaminetetraacetic acid
FBS: Fetal bovine serum
M-CSF: Macrophage colony-stimulating factor
CFUs: Colony-forming units
IFM: Immunofluorescence microscopy
TEM: Transmission electron microscopy
Chek2: Cell cycle checkpoint kinase 2
mtDNA: Mitochondrial DNA
