# Supplementary figures and images for: Activation of the MEK1-CHK2 axis in macrophages by Staphylococcus aureus promotes mitophagy, resulting in a reduction in bactericidal efficacy
Source: Mol Med. 2025 May 29;31:211. doi: 10.1186/s10020-025-01274-7 (PMC12121099; doi:10.1186/s10020-025-01274-7)

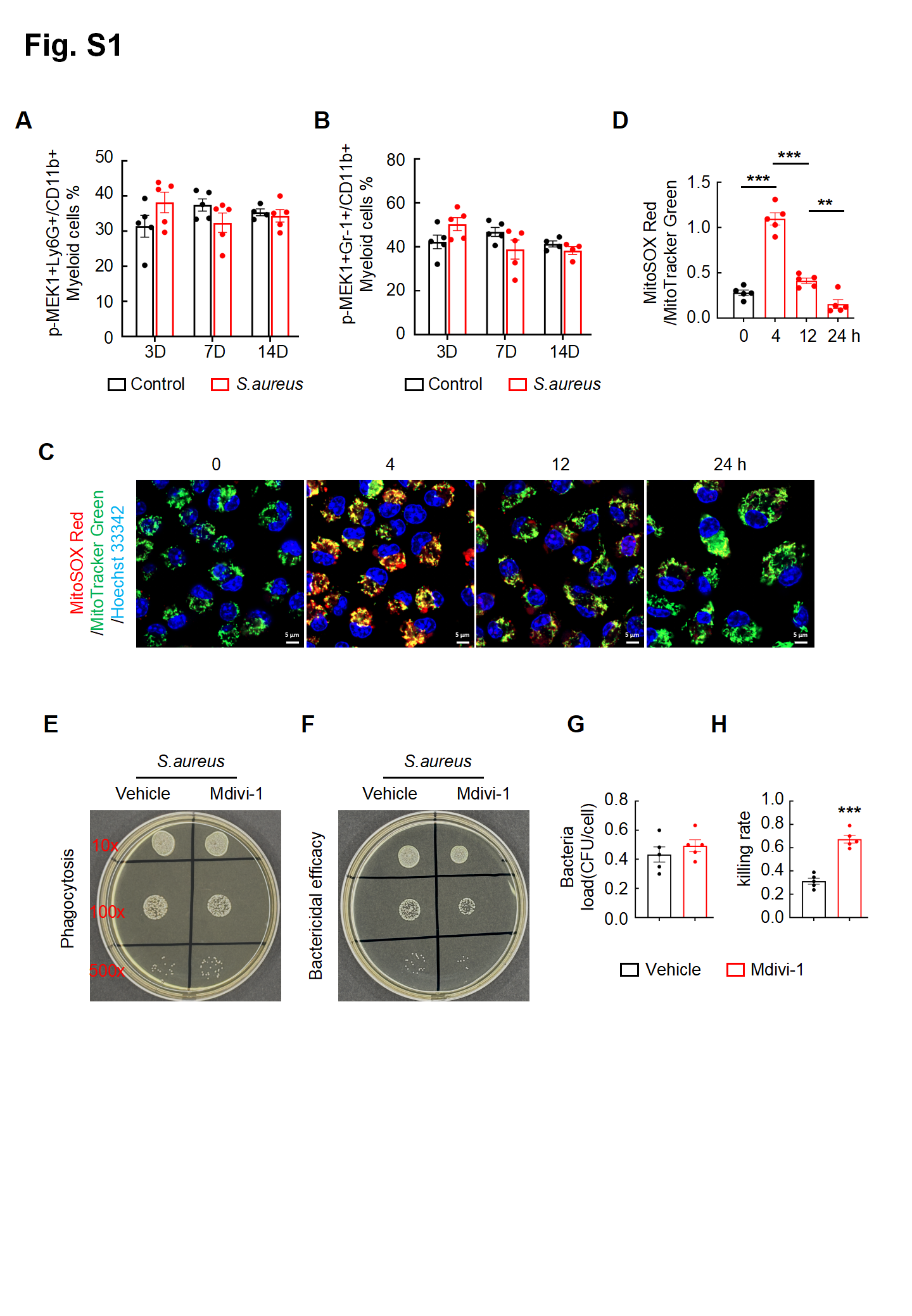

Supplement: Supplementary file 1 — Supplementary Material 1. [file 10020_2025_1274_MOESM1_ESM.tif]
